# 6-Phosphogluconate dehydrogenase inhibition arrests growth and induces apoptosis in gastric cancer via AMPK activation and oxidative stress

**DOI:** 10.1515/biol-2022-0514

**Published:** 2023-02-23

**Authors:** Cheng Chen, Pan Du, Zhenguo Zhang, Di Bao

**Affiliations:** Department of Oncology, Xiangyang Central Hospital, Affiliated Hospital of Hubei University of Arts and Sciences, Xiangyang 441021, China; Institute of Oncology, Hubei University of Arts and Science, Xiangyang 441021, China; Department of Gastroenterology, Xiangyang Central Hospital, Affiliated Hospital of Hubei University of Arts and Sciences, Xiangyang 441021, China

**Keywords:** gastric cancer, 6-PGDH, redox homeostasis, AMPK, lipid biosynthesis

## Abstract

Poor outcomes in advanced gastric cancer necessitate alternative therapeutic strategies. 6-Phosphogluconate dehydrogenase (6-PGDH), an enzyme that catalyzes the decarboxylation step in the oxidative pentose phosphate pathway, has been identified as a promising therapeutic target in many cancers. In this study, we systematically investigated the expression and function of 6-PGDH in gastric cancer. We found that 6-PGDH expression and activity were aberrantly elevated in gastric cancer tissues compared to their adjacent normal tissues. 6-PGDH knockdown using two independent shRNAs resulted in minimal 6-PGDH levels and activity, decreased growth, and enhanced gastric cancer cell sensitivity to 5-flurorouracil. However, 6-PGDH knockdown did not affect the cancer cells. Mechanistic studies showed that 6-PGDH inhibition disrupted lipid biosynthesis and redox homeostasis in gastric cancer, inhibited growth, and induced apoptosis. Notably, the *in vitro* findings were validated using an *in vivo* gastric cancer xenograft mouse model. This study established that 6-PGDH is broadly elevated in gastric cancer patients and that 6-PGDH inhibition can sensitize gastric cancer cells in response to chemotherapy.

## 1 Introduction

Gastric cancer is the second most common cause of cancer-related death worldwide [1]. Treatment options for patients with gastric cancer include gastrectomy and chemotherapy [2]. 5-Flurorouracil (5-FU), alone or in combination with other conventional therapies, is the standard chemotherapy regimen. However, resistance, either acquired or primary, is the main cause of treatment failure [3]. The mechanisms of gastric cancer pathogenesis and chemoresistance are not clear and rely on multiple factors, such as deregulation of developmental pathways and cancer stem cell signaling (Wnt/β-catenin, Hedgehog, Notch, STAT3, and epithelial–mesenchymal transition) [4,5]. Recent studies have suggested altering the biosynthetic metabolism in gastric cancer, including anabolic biosynthesis and redox homeostasis, which might represent a promising therapeutic target.

Reduced levels of nicotinamide adenine dinucleotide phosphate (NADPH) is critical for redox defense and is the driving force of most biosynthetic enzymatic reactions, including DNA and lipids [6]. NADPH production is mediated by glutamine metabolism and the oxidative pentose phosphate pathway (PPP) [7]. 6-Phosphogluconate dehydrogenase (6-PGDH), a key enzyme in the oxidative PPP, is aberrantly activated and plays an important role in a variety of cancers. The repression of 6-PGDH results in altered metabolism, reduced cell growth and survival, and elevated reactive oxygen species (ROS) levels in colon, breast, liver, and thyroid cancers. Recent studies have also highlighted that 6-PGDH inhibition is selective for targeting cancer cells and reversing cancer resistance to chemotherapy [8–12].

In line with previous efforts, this work investigated the expression pattern and functions of 6-PGDH in gastric cancer, and attempted to determine the underlying mechanisms using patient samples and pre-clinical models *in vitro* and *in vivo*.

## 2 Materials and methods

### 2.1 Tissue specimens and cell culture

Surgical tissues from gastric cancer patients were used in this study. The frozen tissue specimens were used for RNA and protein extraction. Formalin-fixed, paraffin-embedded tissue specimens were used for immunohistochemical analysis. Two human gastric cancer cell lines, AGS and MKN-45, were obtained from the Cell Bank of the Type Culture Collection of the Chinese Academy of Sciences. AGS and MKN-45 cells were cultured in RPMI1640 and dulbecco’s modified eagle medium (Gibco, USA) supplemented with 10% fetal bovine serum (FBS) (Gibco, USA), respectively.


**Informed consent:** Informed consent has been obtained from all individuals included in this study.
**Ethical approval:** The research related to human use has been complied with all the relevant national regulations, institutional policies and in accordance with the tenets of the Helsinki Declaration, and has been approved by the ethics committee of Xiangyang Central Hospital (No. 2016-018).

### 2.2 Immunohistochemistry and western blot analysis

Tissue blocks were sectioned, dewaxed in xylene, and rehydrated prior to antigen retrieval using citrate buffer. The tissue sections were then washed with phosphate buffered saline (PBS), incubated with 1:500 diluted anti-6-PGDH antibody (Thermo Fisher Scientific #PA5-25362), the designated secondary antibody, and 3,3-diaminobenzidine, according to the standard staining protocol. Cell nuclei were counterstained with a hematoxylin solution (Sigma-Aldrich, USA). Immunostaining experiments were analyzed independently by two pathologists. Under a microscope (Zeiss, Germany), 6-PGDH protein expression was measured by multiplying the staining area by the intensity fraction of four random regions per section. The percentage of positive cells was scored as 1 (1–25%), 2 (26–50%), 3 (51–75%), or 4 (76–100%), and the staining intensity was scored as 0, 1, 2, or 3. Staining scores <6 and ≥6 were considered as low and high expression, respectively. For western blot analysis, cells were lysed using RIPA buffer (Thermo Fisher Scientific). Proteins were loaded and resolved using sodium dodecyl sulfate–polyacrylamide gel electrophoresis and analyzed by western blot using standard protocol. The primary antibodies used are listed as follows (1:1,000 dilution): anti-caspase 3 (ab32351, Abcam), anti-cleaved caspase 3 (ab32042, Abcam), anti-MIC-1 (8479, CST), anti-Bim (2933, CST), anti-β-actin (3700, CST), anti-p-AMPK (9158, CST), anti-AMPK (2537, CST), anti-ACC1 (4190, CST), anti-p-ACC1 (11818, CST), and anti-Nox2 (ab129068, Abcam). Rabbit secondary antibody (7074, CST, 1:2,000 dilution) and mouse secondary antibody (14709, CST, 1:2,000 dilution) were purchased from cell signaling. The molecular weight reflecting the location of target protein bound with its antibody was indicated in the figures.

### 2.3 6-PGDH enzyme activity assay

6-PGDH enzyme activity was assessed using total cell or tissue lysates and measured using a 6-PGDH assay kit (Abcam, #ab241016), according to the manufacturer’s instructions.

### 2.4 Real-time PCR

Total RNA from the tissue specimens was extracted using an mRNA isolation kit (Merck, #11741985001). The RNA concentration was determined spectrophotometrically at 260 nm. The reverse transcription reaction was performed using the iScript cDNA Synthesis Kit (Bio-Rad, #1708891). Quantitative real-time PCR was performed using the SYBR Green Supermix (Bio-Rad, #1708880). The sequences of the exon–exon junction primers for 6-PGDH were ATT CTC AAG TTC CAA GAC ACC G (forward) and GTG GTA AAA CAG GGC ATG GGA (reverse) [13].

The primers for β-actin are CTC CAT CCT GGC CTC GCT GT(forward) and GCT GTC ACC TTC ACC GTT CC (reverse). The primer concentrations were 10 mM. The specific reaction system is to add 5 µL of sybgreen, 1 µL of forward primer, 1 µL of reverse primer, and 3 µL of cDNA. The reaction thermal profiles were reaction at 50°C for 2 min (one time) and pre-denaturation at 95°C for 2 min (one time). Denaturation at 95°C for 15 s followed by annealing and extension at 60°C for 30 s (repeated for 40 cycles). The mRNA levels of 6-PGDH were quantified using a comparative CT method with β-actin levels for normalization.

### 2.5 Stable knockdown of 6-PGDH

A stable knockdown of endogenous human 6-PGDH was achieved using a lentiviral vector. pGIPZ shRNA 6-PGDH A was generated using 5′-AGG ACT GTC TCC AAA GTT G-3′, and pGIPZ shRNA 6-PGDH B was generated with 5′-GGT GGA TGA TTT CAT CGA GAA ACT CG-3′ targeting the coding region of the 6-PGDH transcript. The 6-PGDH-depleted AGS and MKN-45 cells were established by strictly following the protocol provided by Dharmacon GIPZ lentiviral shRNA Transfection Starter Kit and validated using western blot analysis.

### 2.6 Proliferation and DNA damage assays

For the proliferation assay, cells were seeded in a 96-well plate. After 24 h of 5-FU (Sigma, F6627) treatment, cell proliferation was determined using a BrdU Cell Proliferation Assay Kit (Abcam, #ab126556). DNA damage was determined using the DNA Damage Quantification Colorimetric Kit (Abcam, #ab284577), followed by absorbance measurements on a microplate reader.

### 2.7 Migration assay

Cell migration was assessed using the CytoSelect 24-well Cell Migration Assay Kit (Cell Biolabs Inc. #CBA-100). Briefly, 10,000 cells together with 5-FU in culture medium without FBS, were seeded into the insert of a transwell plate. The culture medium containing 10% FBS was placed in the lower chamber. After 6 h, the cells that spread on the upper surfaces of the filter (non-migrated cells) were wiped away using cotton swabs. The insert was stained, and the migrated cells were counted under a microscope.

### 2.8 Measurement of the NADPH/NAD+ ratio, ROS, and lipids

NADPH/NAD+ ratio was determined by measuring NADPH/NADP+ concentrations according to the protocol (BioAssay Systems, #ECNP-100). Briefly, NADPH and NADP+ were extracted from the cells and measured at 565 nm based on a glucose dehydrogenase cycling reaction, in which the formed NADPH reduces a formazan (MTT) reagent. The intensity of the product color is proportionate to the NADPH/NADP+ concentrations. The samples were then transferred to 96 plates for measuring the absorbance of 450 nm. The amount of oxidized NAD (NAD+) was presented subtracting NADH from NAD_total_. The ratio of NAD/NADH in a sample was calculated by the equation: ratio = (NAD_total_ – NADH)/NADH. Intracellular ROS was measured using OxiSelect™ Intracellular ROS Assay Kit (Cell Biolabs, #STA-342). The amount of intracellular ROS was measured by detecting dichlorodihydrofluorescein, which is the cleavage product of carboxy-H_2_DCFDA by ROS. In all, cells were seeded in a plate. After 24 h of seeding, cells were washed with PBS and loaded with 10 mM carboxy-H_2_DCFDA for 30 min. The cells were harvested and resuspended in PBS. The mixtures were incubated at 30°C for 2 h. Cells were fixed by 2% paraformaldehyde. Untreated group served as control. Data were analyzed using Flow Jo Software (Version 10.1). According to the manufacturer’s instructions, the manufacturer’s Lipid Quantification Kit (Cell Biolabs, #STA-613) was used to quantify the lipids. The lipids in the tissue were extracted before total lipid measurements. The Lipid Quantification Kit’s enzymatic processes produced a dye that absorbs light at 540 nm. Each individual assay’s standard curve was created using the serial dilutions of the standard solution included in each kit. Create a standard curve and use the optical density values at specific absorbance wavelengths to best fit the corresponding standard concentrations, with *R* > 0.99 as the established standard for a linear function.

### 2.9 Xenograft mouse model

All animal experiments were approved by protocol No. 201706 and were performed in accordance with the guidelines of the Institutional Animal Care and Use Committee. Mice were housed at the Animal Center of Hubei University of Arts and Sciences. The cells were injected subcutaneously into irradiated (240 cGy) immunodeficient mice. For a fair comparison, we inoculated the same mice with shRNA control cells on their left flank and shRNA 6-PGDH cells on the right flank. The tumor size was monitored for 4 weeks. When the tumor reached 1,000 mm^3^, the mice were euthanized using the gradual-fill method of CO_2_ euthanasia (the CO_2_ flow rate displaced 10–30% of the cage volume per minute). Tumors were dissected and tumor proteins were extracted using RIPA buffer.


**Ethical approval:** The research related to animal use has been complied with all the relevant national regulations and institutional policies for the care and use of animals, and has been approved by the Institutional Animal Care and Use Committee of the Hubei University of Arts and Sciences (approval no. 2021074689).

### 2.10 Statistical analyses

Results were obtained from a minimum of three independent experiments in triplicate and are presented as mean ± standard deviation. Statistical analyses for comparisons of two categorical variables were conducted using the Mann–Whitney *U-*test. To ascertain each independent factor, one-way analysis of variance and subsequently unpaired Student’s *t*-tests were also conducted. Statistical significance was set at *p* < 0.05.

## 3 Results

### 3.1 Aberrant activation of 6-PGDH in gastric cancer

**Figure 1:**
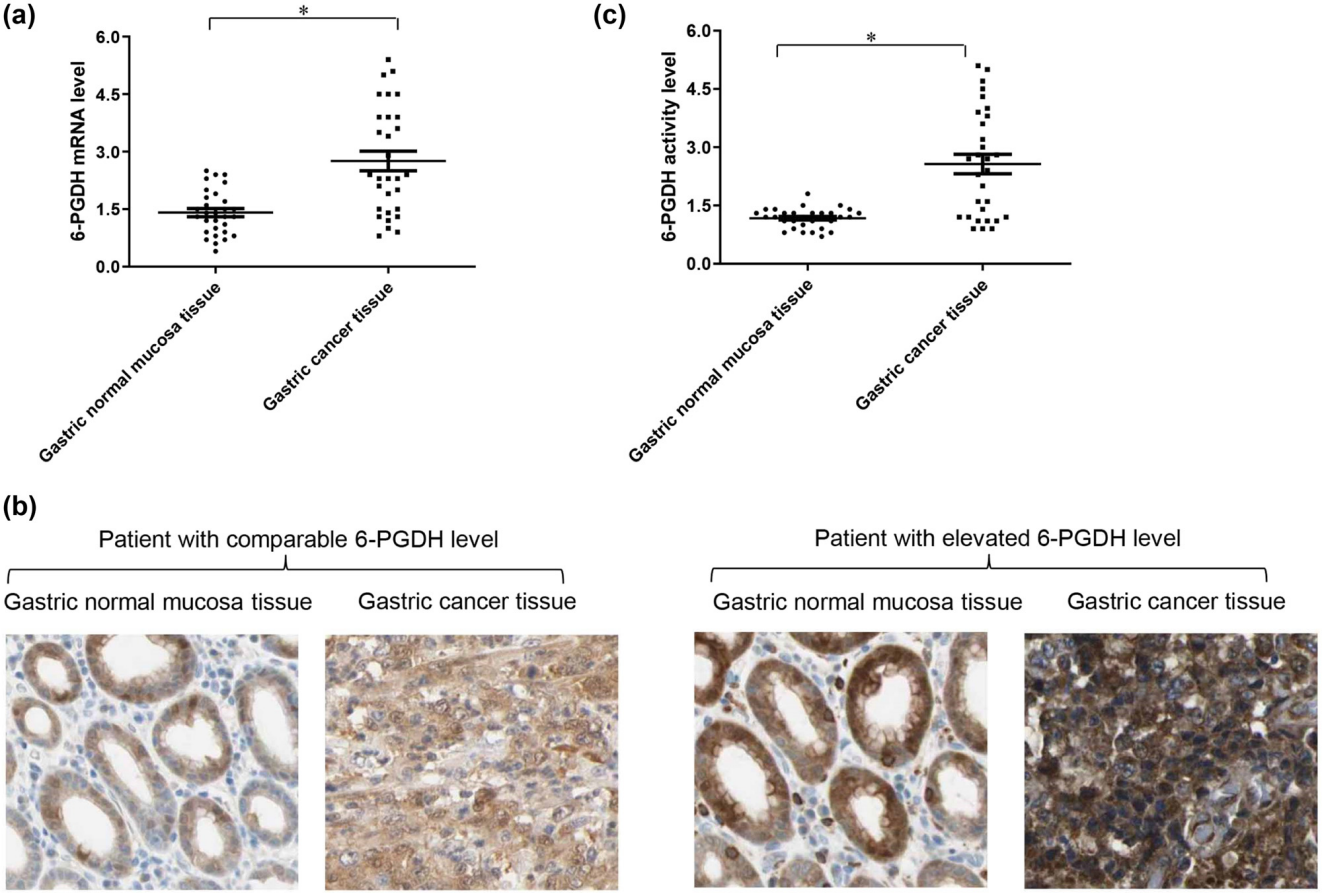
6-PGDH expression and activity are elevated in patients with gastric cancer. Scatter plot of mRNA (a), and enzyme activity (c) of 6-PGDH in gastric cancer tissues and adjacent normal gastric tissue obtained from gastric cancer patients (*n* = 30). The average expression and activity of 6-PGDH is significantly increased in gastric cancer tissue compared to normal gastric normal tissue. (b) Representative images of 6-PGDH immunohistochemistry in one patient with comparable 6-PGDH level between normal and cancer tissue and another patient with elevated 6-PGDH level in cancer tissue. Scale bar is 50 μm. * *p* < 0.05, compared to normal.

To identify the 6-PGDH expression pattern in normal gastric tissues and gastric cancer tissues, we used real-time PCR and immunohistochemistry approaches to determine the mRNA transcript and protein levels in 30 paired gastric cancer and adjacent normal gastric tissues obtained from subjects at different stages of disease (Table S1). Overall, we found that the average mRNA and protein levels of 6-PGDH were significantly increased by two-fold in gastric cancer tissues compared to normal gastric tissues (Figure 1a and b). However, 6-PGDH expression in individual patient samples demonstrated that ∼77% (23 of 30) of the tested patients had significantly elevated 6-PGDH expression in cancer tissues and ∼23% (7 out of 30) had comparable 6-PGDH expression levels between paired cancer and normal gastric tissues (Figure 1b and Table S2). In addition, 6-PGDH expression varied between normal and cancerous tissues. The highest fold-change between paired normal and cancerous gastric tissues was 3.5 (Table S2). We further found that increased expression was associated with increased enzyme activity (Figure 1c and Table S2). Furthermore, we found a significant positive correlation between the mRNA, protein, and activity levels of 6-PGDH (Figure S1) A total of 109 gastric cancer specimen cohorts were used to estimate 6-PGDH expression by immunohistochemistry staining. Overall survival (OS) is defined as the time from the end of the first operation to death. Time in therapeutic range (TTR) is defined as the time from the end of the first operation to the first recurrence. The OS and TTR for two subgroups (6-PGDH^low^ and 6-PGDH^high^) are presented in Figure S2. Here, we found that the 6-PGDH^high^ subgroup had a significantly shorter OS (*p* < 0.001) and TTR (*p* < 0.001) than the 6-PGDH^low^ groups.

### 3.2 Functional effects of 6-PGDH in gastric cancer

**Figure 2:**
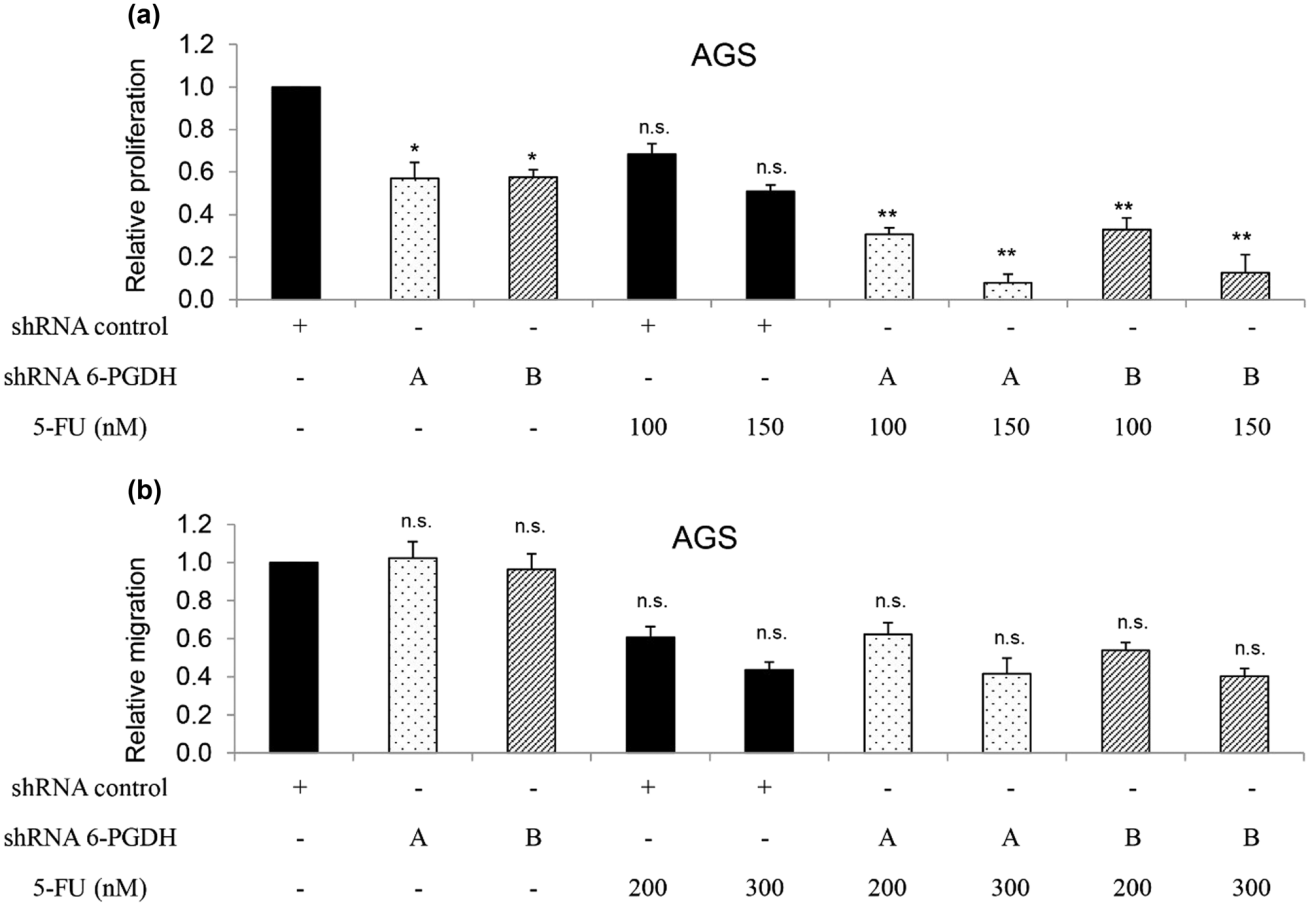
6-PGDH knockdown inhibits gastric cancer cell growth and augments 5-FU’s inhibitory effects. (a) AGS cells with 6-PGDH knockdown displayed decreased proliferation and further growth inhibition after 5-FU treatment compared to control cells. (b) 6-PGDH knockdown did not affect AGS cell migration. The experiments were performed at least three times with triplicates. Proliferation was analyzed after 24 h drug treatment. Migration was analyzed after 6 h drug treatment. * *p* < 0.05, compared to shRNA control. ** *p* < 0.05, compared to 5-FU.

**Figure 3:**
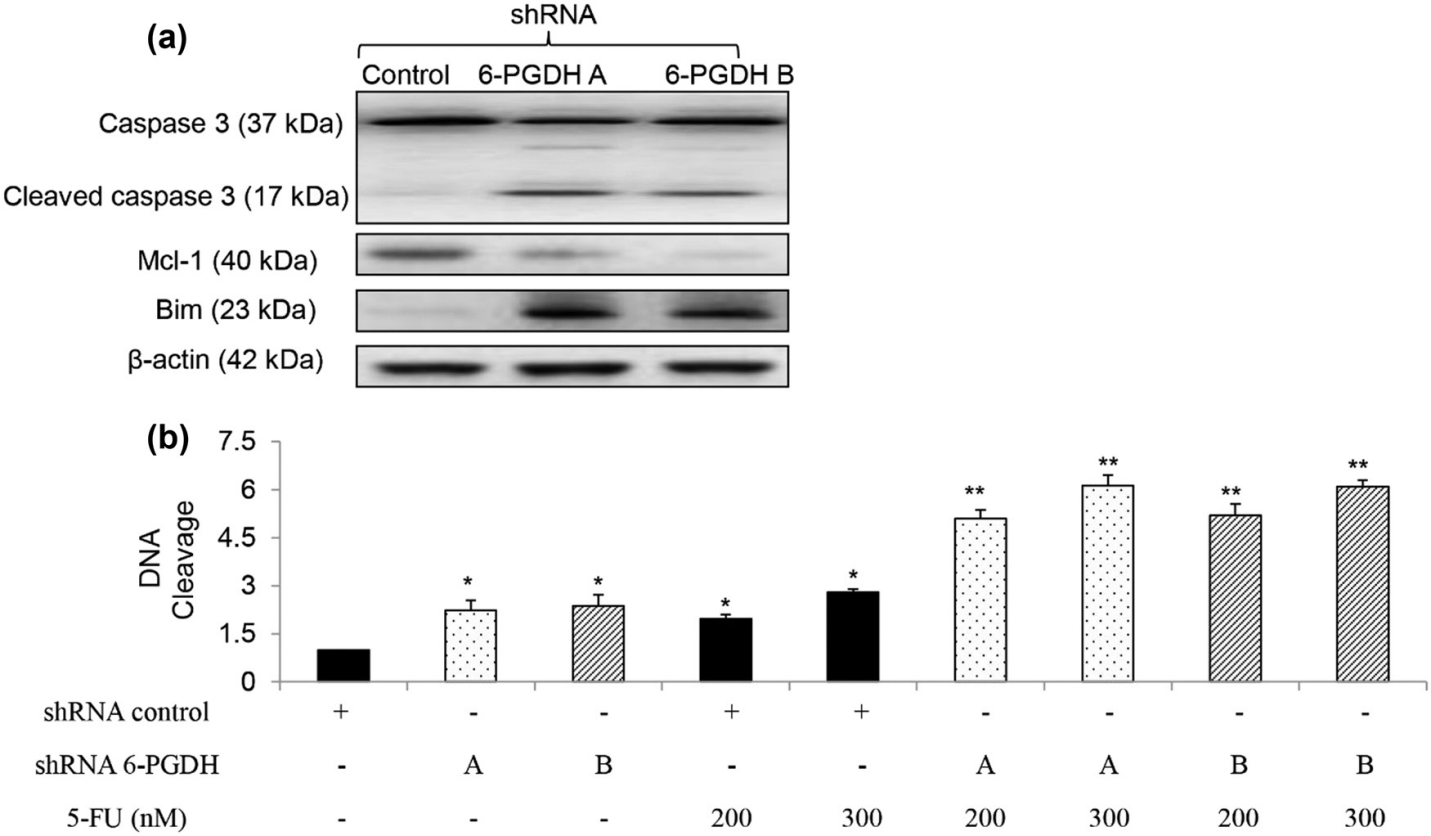
6-PGDH knockdown induces gastric cancer cell apoptosis and augments 5-FU’s inhibitory effects. (a) Representative western blot image showing the decreased caspase 3, increased cleaved caspase 3, decreased Mcl-1, and increased Bim in 6-PGDH-depleted AGS cells. Western blot was performed at 72 h post-transfection. AGS cells with 6-PGDH knockdown displayed decreased proliferation and further growth inhibition after 5-FU treatment compared to control cells. (b) AGS cells with 6-PGDH knockdown displayed increased DNA damage compared to control group. The experiments were performed at least three times. * *p* < 0.05, compared to shRNA control. ** *p* < 0.05, compared to 5-FU.

To identify the functional effects of 6-PGDH on gastric cancer, we analyzed the growth, migration, and survival of gastric cancer cells after stable knockdown of 6-PGDH using two independent shRNAs. We also analyzed the sensitivity of 6-PGDH-depleted gastric cancer cells to 5-FU, which is the first-line chemotherapeutic agent for advanced gastric cancer [14]. AGS cells transduced with a lentiviral vector harboring 6-PGDH shRNA showed minimal levels of 6-PGDH expression and activity (Figure S3) and reduced cell proliferation (Figure 2a). In addition, 6-PGDH knockdown significantly enhanced the inhibitory effects of 5-FU on cell growth (Figure 2a). In contrast, 6-PGDH knockdown did not affect AGS cell migration or 5-FU sensitivity (Figure 2b). Of note, anti-proliferation and sensitization to chemotherapy induced by 6-PGDH depletion was not limited to AGS cells; MKN-45 responded in a similar manner (Figure S4a and b). Next, we analyzed apoptosis and DNA damage in the 6-PGDH-depleted cells. We found that 6-PGDH knockdown decreased caspase-3 and increased cleaved caspase-3 levels (Figure 3a), indicating that 6-PGDH knockdown induced apoptosis in gastric cancer cells. We observed decreased levels of anti-apoptotic protein Mcl-1 and increased levels of pro-apoptotic protein Bim (Figure 3a). Consistent with this finding, 6-PGDH depletion significantly increased DNA damage (Figure 3b, Figure S4c). In addition, the pan-caspase inhibitor Z-VAD-FMK significantly reversed DNA damage induced by 6-PGDH depletion in gastric cancer cells (Figure S5). These results clearly demonstrated increased apoptosis and DNA damage in gastric cancer cells after 6-PGDH inhibition. Notably, 6-PGDH inhibition further augmented the apoptosis and DNA damage induced by 5-FU. Similar results were obtained in MKN-45 cells with stable 6-PGDH knockdown, suggesting general functional effects of 6-PGDH on gastric cancer growth, survival, and chemosensitivity, but not migration.

### 3.3 6-PGDH inhibition disrupts biosynthesis and redox homeostasis in gastric cancer

**Figure 4:**
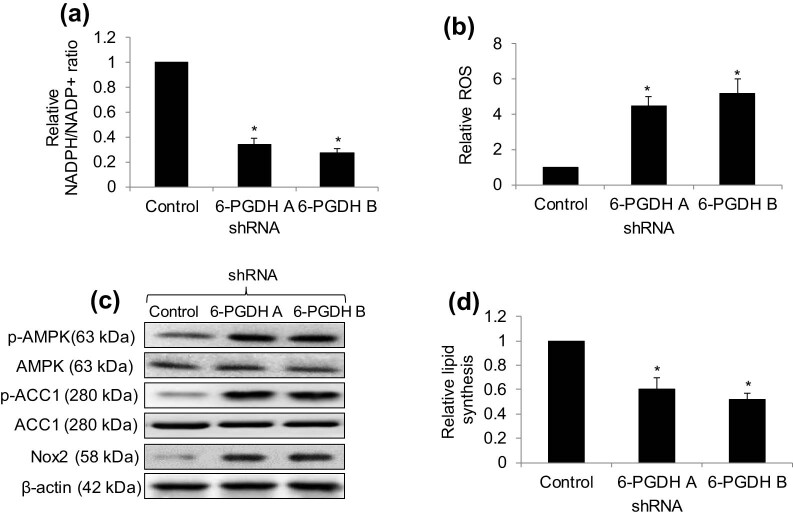
6-PGDH knockdown disrupts biosynthesis and redox homeostasis in gastric cancer cells. 6-PGDH knockdown significantly decreased NADPH/NADP+ ratio (a) and increased ROS (b) in AGS cells. (c) Representative western blot showing the increased Nox2, p-AMPK, and p-ACC1 level in 6-PGDH-depleted AGS cells. (d) 6-PGDH knockdown significantly decreased lipid synthesis in AGS cells. * *p* < 0.05, compared to shRNA 6-PGDH.

**Figure 5:**
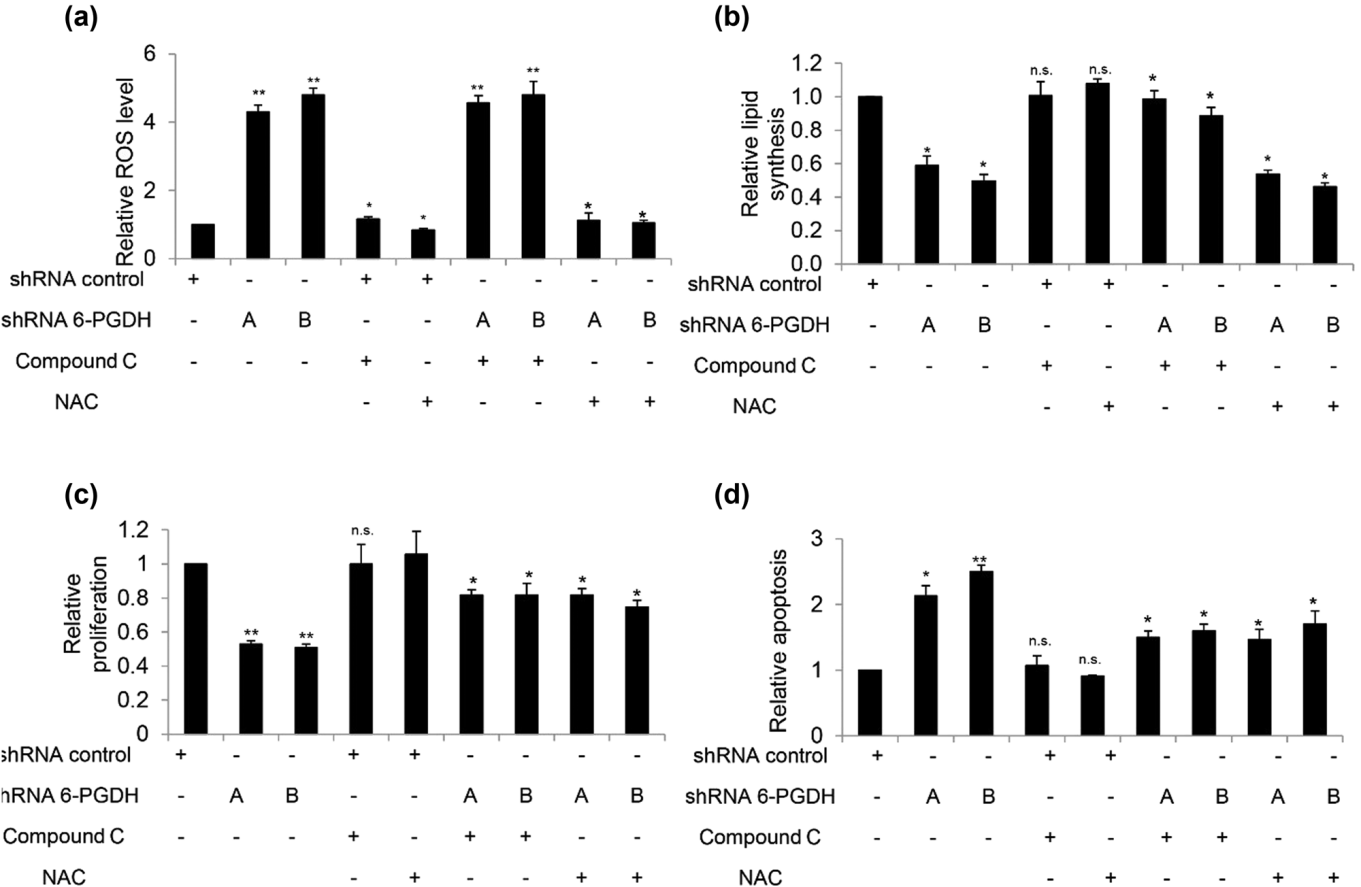
NAC and compound C reverse the decreased lipid biosynthesis and increased oxidative stress in 6-PGDH-depleted gastric cancer cells. (a) Addition of NAC but not compound C significantly rescued increased ROS in 6-PGDH-depleted cells. (b) Addition of compound C but not NAC significantly rescued decreased lipid level in 6-PGDH-depleted cells. Addition of NAC and compound C significantly rescued decreased growth (c) and increased apoptosis (d) in 6-PGDH-depleted cells. The experiments were performed at least three times with duplicates or triplicates. NAC at 3 mM and compound C at 10 μM were used. * *p* < 0.05, compared to shRNA 6-PGDH.

To understand the underlying mechanisms of the 6-PGDH functional effects, we first assessed the NADPH/NADP+ ratio in 6-PGDH knockdown cells, as 6-PGDH is the major source of NADPH [15]. Not surprisingly, we observed a decreased NADPH/NADP+ ratio in 6-PGDH knockdown gastric cancer cells (Figure 4a), suggesting a decreased intracellular redox potential. Consistently, we found that 6-PGDH knockdown significantly increased ROS (Figure 4b) and NADPH oxidase 2 (Nox2) levels (Figure 4c and Figure S6). In addition, increased phosphorylation of AMP-activated protein kinase (AMPK) at T172 and acetyl-CoA carboxylase 1 (ACC1) at S79, and decreased lipid levels were detected in 6-PGDH knockdown cells (Figure 4d). This demonstrates that 6-PGDH knockdown leads to AMPK activation and a subsequent reduction of lipid synthesis. Addition of the anti-oxidant n-acetyl-L-cysteine (NAC) significantly reduced ROS levels in 6-PGDH knockdown cells but did not rescue the decreased lipid levels (Figure 5a and b). In contrast, addition of the AMPK inhibitor compound C significantly increased lipid levels in 6-PGDH knockdown cells but did not rescue increased ROS levels. Notably, the addition of NAC or compound C to 6-PGDH knockdown cells significantly rescued the decreased cell proliferation and increased cell apoptosis (Figure 5c and d). These results clearly demonstrate that 6-PGDH inhibition disrupts biosynthesis and redox homeostasis in gastric cancer cells by inhibiting growth and inducing apoptosis.

### 3.4 6-PGDH inhibition arrests gastric cancer growth and disrupts biosynthesis and redox homeostasis in an *in vivo* mouse model

**Figure 6:**
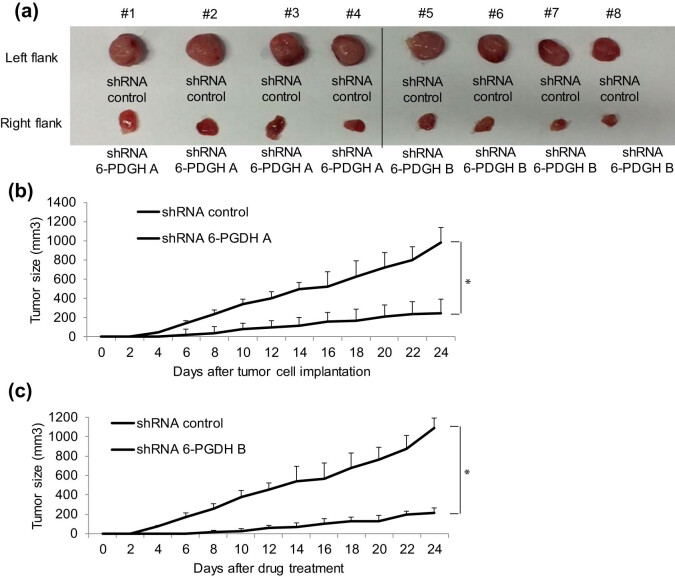
6-PGDH knockdown inhibits gastric cancer growth *in vivo*. (a) Images showing dissected tumors in mice injected with AGS cells with 6-PGDH knockdown. Two stable AGS cell lines with 6-PGDH knockdown (b and c) displayed significantly growth arrest in mice. We inoculated the same mice with shRNA control cells on its left flank and shRNA 6-PGDH cells on its right flank. Therefore, both shRNA 6-PGDH A and B have their own shRNA control, respectively. * *p* < 0.05, compared to shRNA control.

**Figure 7:**
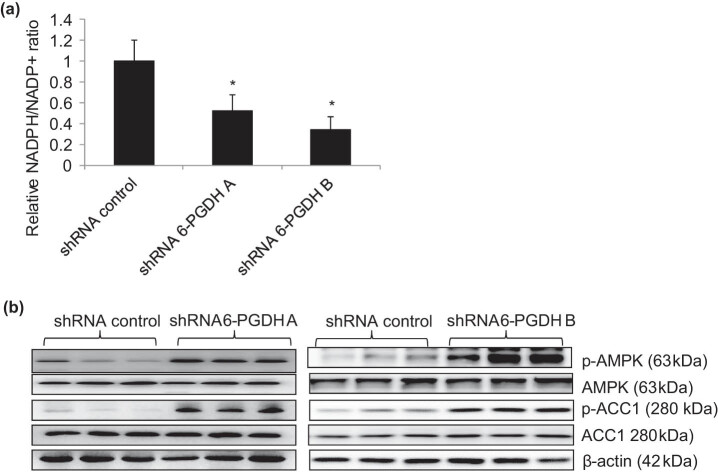
6-PGDH knockdown disrupts biosynthesis and redox homeostasis *in vivo.* 6-PGDH shRNA tumors display decreased NADPH/NADP+ ratio (a) and increased p-AMPK and p-ACC1 level (b). * *p* < 0.05, compared to shRNA control.

To investigate whether the data obtained using the cell culture system were reproducible *in vivo*, we performed functional and mechanistic studies using a xenograft tumor model. We subcutaneously injected 6-PGDH knockdown cells into immunodeficient mice and monitored tumor growth for ∼4 weeks. We observed a significantly smaller size and the delayed growth of tumors formed by 6-PGDH knockdown cells compared to the control (Figure 6). In line with our *in vitro* data, we observed a significant decrease in the NADPH/NADP+ ratio in 6-PGDH knockdown tumors compared to that in the control (Figure 7a). Western blot analysis of tumor lysates demonstrated increased p-AMPK and p-ACC1 levels (Figure 7b), suggesting increased AMPK activation in 6-PGDH knockdown tumors. Taken together, we demonstrated that 6-PGDH inhibition arrests gastric cancer growth and disrupts biosynthesis and redox homeostasis *in vivo*.

## 4 Discussion

Although previously unstudied in gastric cancer, substantial evidence has emphasized the important roles of 6-PGDH in multiple aspects of cancer cells, from cancer initiation to metastasis, and resistance to drug treatment [16], suggesting an interesting therapeutic target for the investigation of gastric cancer. Indeed, our findings are consistent with previous reports and provide the first evidence that 6-PGDH is overexpressed and hyperactivated in most gastric cancer patients, and that 6-PGDH inhibition is active against gastric cancer by disrupting lipid biosynthesis and redox homeostasis.

6-PGDH expression and activity have been reported to be elevated in cancer cells compared to normal cells [17,18], and cancer cells after prolonged treatment of chemotherapy drugs [19]. Focusing on 30 paired gastric normal and cancer tissue samples, we found that the mRNA and protein levels of 6-PGDH were increased two-fold in gastric cancer tissues regardless of disease stage, age, tumor sites, and histology (Figure 1 and Table S1). Notably, 6-PGDH hyperactivation occurred in most, but not all, gastric cancer patients (Figure 1b, Table S2), suggesting that 6-PGDH hyperactivation is not a universal feature but only present in a particular group of patients with gastric cancer. Four major mechanisms that regulate 6-PGDH hyperactivation in cancer have been reported: YTH domain family 2 promotes 6-PGDH mRNA translation by directly binding to the m6A modification site [20], nuclear factor erythroid 2-related factor 2 activates 6-PGDH via a well-conserved anti-oxidant response element [21], epidermal growth factor receptor phosphorylates and activates 6-PGDH by Fyn [18], and direct structural interactions with malic enzyme1 [22]. These factors are worth investigating to further understand the regulation of 6-PGDH in gastric cancer.

We next showed that 6-PGDH hyperactivation was correlated with gastric cancer progression. In particular, loss-of-function studies demonstrated that growth and survival, but not migration stimulation, play prominent roles with 6-PGDH in gastric cancer (Figures 2 and 3). This is supported by previous findings that 6-PGDH inhibition suppresses proliferation and induces cell death in leukemia, ovarian, and breast cancers [23–25]. Although 6-PGDH promotes migration and invasion of lung and cervical cancer cells [9,26], 6-PGDH inhibition did not affect the migration of gastric cancer cells (Figure 2b). The important role of 6-PGDH in gastric cancer growth was clearly demonstrated in a xenograft mouse model (Figure 6). In addition, 6-PGDH protected gastric cancer cells from chemotherapy-induced toxicity (Figures 2 and 3), which is consistent with the reported roles of 6-PGDH in the development of chemoresistance in cervical, lung, and ovarian cancers [19,25]. Selective and potent pharmacological inhibitors of 6-PGDH, such as physcion and S-nitrosation, have been developed, and their anticancer activities have been validated in some cancers [27]. Our functional studies using a genetic approach serve as a proof-of-concept to demonstrate the critical role of 6-PGDH in gastric cancer. Further studies should be conducted on the cytotoxic effects of 6-PGDH inhibitors alone and in combination with chemotherapy against gastric cancer, along with the toxicological profiles of these inhibitors. In addition to the antitumor effect of 6-PGDH inhibitors, their toxicity to normal tissues should also be seriously considered in clinical practice. 6-PGDH is a crucial PPP enzyme involving the production of NADPH that enables cells to combat the oxidative stress via the glutathione system. It has been reported that excessive oxidative stress can lead to toxicity in liver and kidney [28,29]. Therefore, 6-PGDH inhibitors may cause liver and kidney damage at least when used as anticancer agents. Further experiments should be performed to determine this issue and explore how to decrease the harmful effects of 6-PGDH inhibition on normal tissues.

Consistent with the identified role of 6-PGDH in metabolic reprogramming and redox homeostasis [30] our work showed that 6-PGDH is important for the coordination between lipid biosynthesis and redox homeostasis to promote gastric cancer growth *in vitro* and *in vivo*, at least in part by controlling the intracellular levels of NADPH and activating AMPK (Figures 4 and 5). Overall, our mechanistic studies demonstrated that (1) 6-PGDH knockdown led to AMPK activation and subsequent reduction of lipid synthesis, (2) 6-PGDH knockdown decreased the NADPH/NADP+ ratio, (3) 6-PGDH knockdown resulted in oxidative stress, (4) AMPK activation/lipid biosynthesis disruption and oxidative stress were independent events in 6-PGDH-depleted gastric cancer cells, and (5) both disrupted lipid synthesis and increased oxidative stress contributed to the inhibition of growth and survival by 6-PGDH depletion. Based on the results obtained from rescue studies, we speculate that increased oxidative stress and reduced lipid synthesis play equal roles in inhibiting gastric cancer cells. In addition, we speculate that oxidative stress is a consequence of a decreased NADPH/NADP+ ratio. Previous work demonstrated that 6-PGDH and AMPK coordinate cancer cell metabolism [31], and our work agrees with and further extends the previous work by adding NADPH to the list of cancer cell metabolism regulators. To maintain a high proliferation rate and promote survival during stress, cancer cells are known to be more dependent on [32] supplementation than normal cells for redox hemostasis, lipid oxidation, and biomolecular synthesis. With regards to NADPH, intracellular redox homeostasis plays critical roles in various cellular processes and signaling pathways during carcinogenesis and tumor progression [33]. In particular, the predominant roles of oxidative stress and lipid synthesis are cell growth and survival, rather than migration. This may explain why 6-PGDH inhibition did not affect cell migration. 6-PGDH inhibition decreased the NADPH/NADP+ ratio, increased ROS, activated AMPK, decreased ACC1 activity, and reduced lipid synthesis (Figures 4 and 5), suggesting that 6-PGDH is an attractive therapeutic target for gastric cancer.

In conclusion, our work establishes that 6-PGDH is aberrantly activated in most gastric cancer patients, and that 6-PGDH inhibition is active against gastric cancer *in vivo* by targeting NADPH and AMPK-related cell metabolism. These discoveries are critical preclinical steps toward investigating 6-PGDH pharmacological inhibitors in combination to achieve chemosensitization in gastric cancer.

## Supplementary Material

Supplementary Figure
