# Supplementary material for: 6-Phosphogluconate dehydrogenase inhibition arrests growth and induces apoptosis in gastric cancer via AMPK activation and oxidative stress
Source: Open Life Sci. 2023 Feb 23;18(1):20220514. doi: 10.1515/biol-2022-0514 (PMC9961966; doi:10.1515/biol-2022-0514)
Supplement: Supplementary Figure [file biol-2022-0514-sm.pdf]

Supplementary material

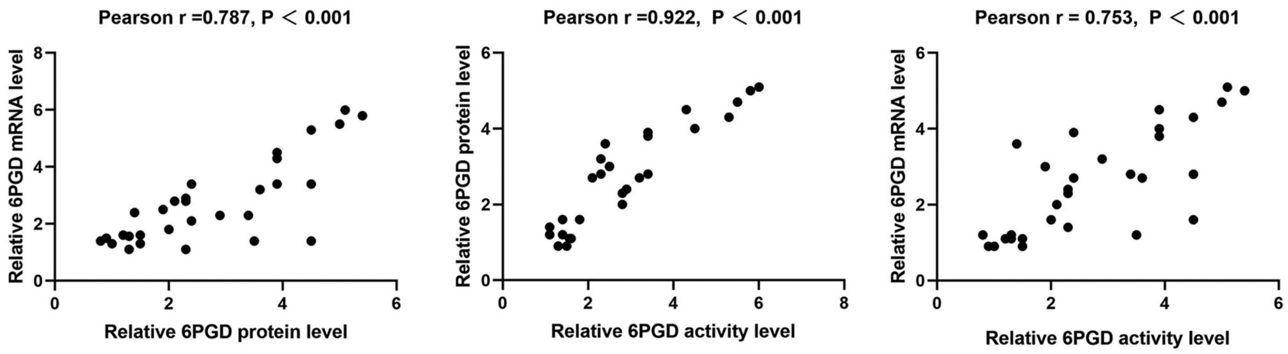

Figure S1: The correlation analysis of 6-PGDH mRNA, protein and activity levels in gastric cancer patients.

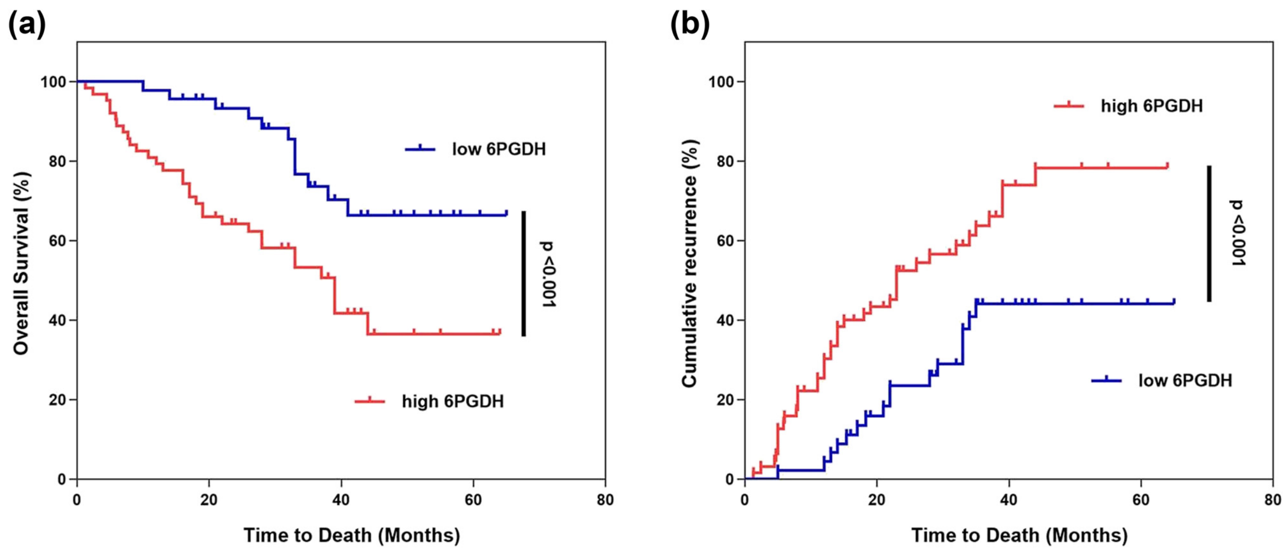

Figure S2: Kaplan–Meier survival analyses based on 6PGD expression in gastric cancer patients. The OS and TTR of postoperative gastric cancer patients based on 6PGD expression. Patients with 6PGD high displayed the shortest OS ($p < 0.001$, log-rank test) and TTR ($p < 0.001$, log-rank test).

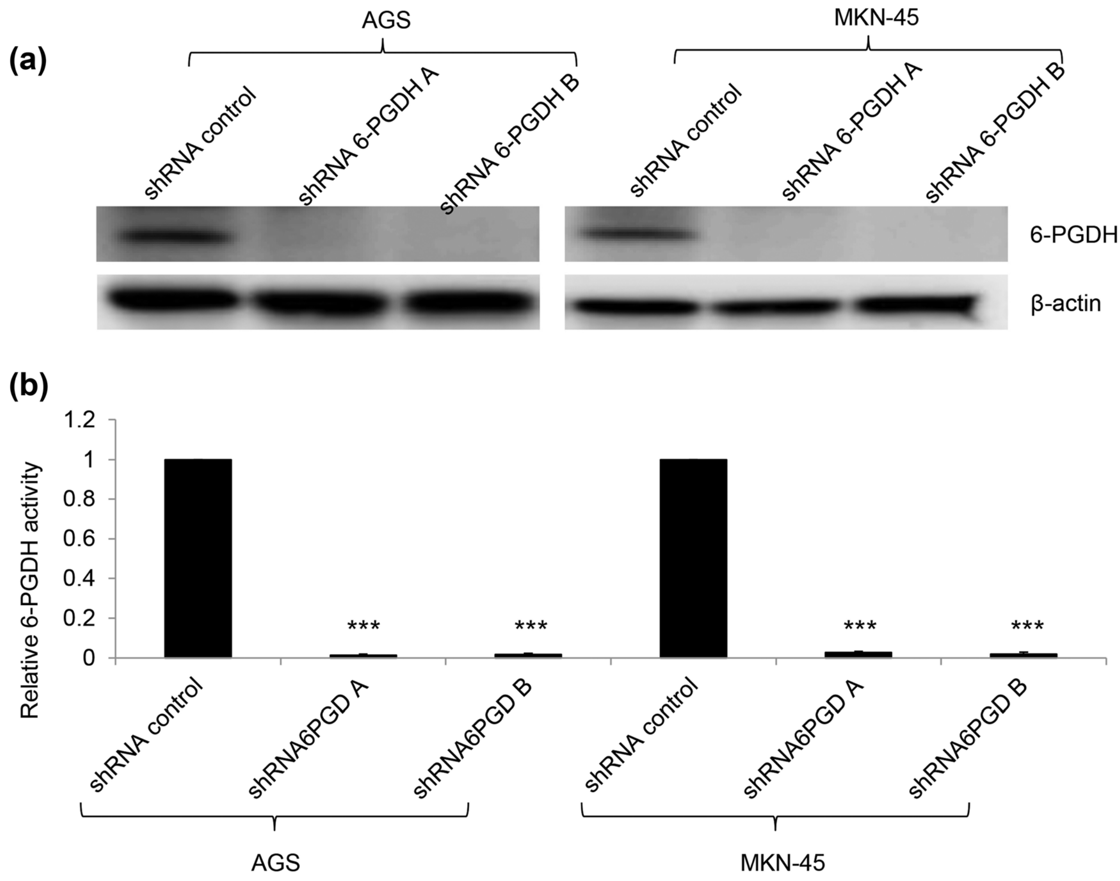

Figure S3: 6PGD inhibition by shRNA results in minimal protein (a) and enzyme activity (b) of 6PGD in AGS and MKN-45 cells. Two independent shRNA were used.

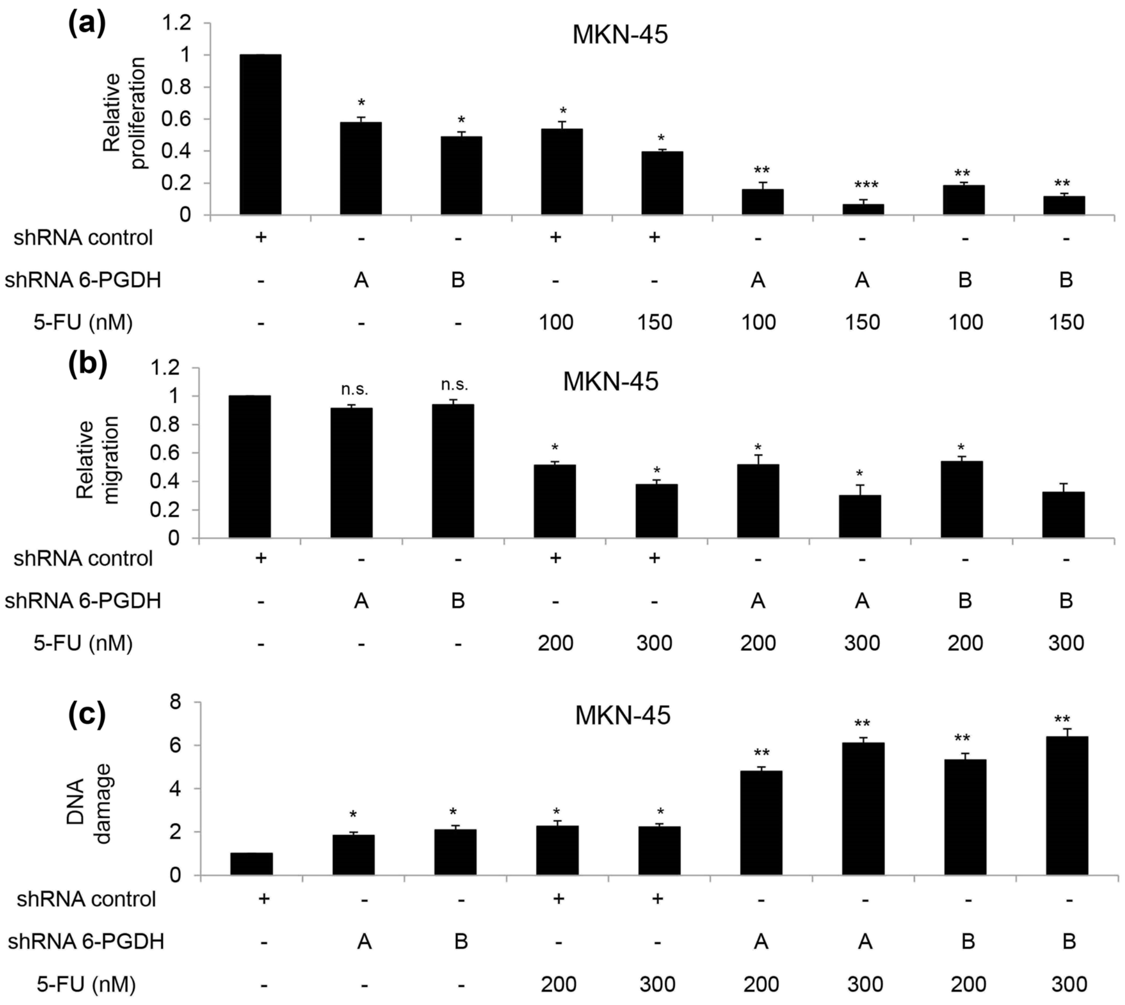

Figure S4: 6PGD knockdown inhibits gastric cancer cell growth and survival, and augments 5-FU's inhibitory effects. (a) MKN-45 cells with 6PGD knockdown displayed decreased proliferation and further growth inhibition after 5-FU treatment compared to control cells. (b) 6PGD knockdown did not affect MKN-45 cell migration. (c) MKN-45 cells with 6-PGDH knockdown displayed increased DNA cleavage compared to control group. * $p < 0.05$, ** $p < 0.05$, *** $p < 0.01$, $n = 3$.

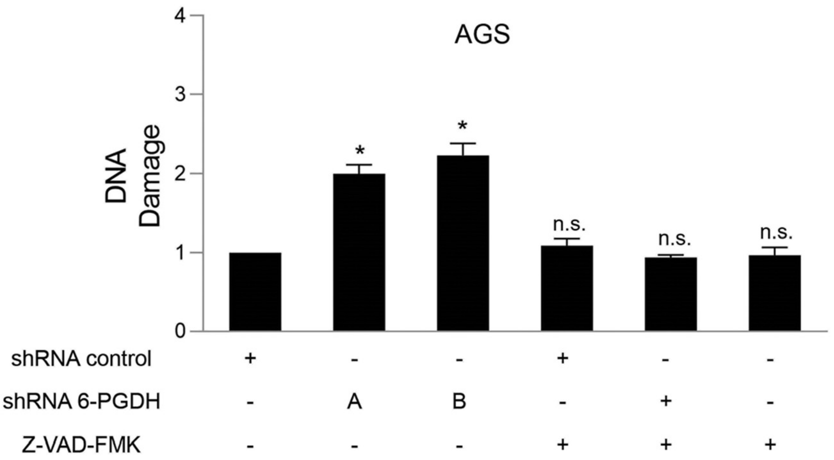

Figure S5: Z-VAD-FMK reverses DNA damage by 6PGD depletion in gastric cancer cells. Z-VAD-FMK at 10 μM was added to the cells. * $p <$ 0.05, compared to shRNA control, $n = 3$.

Table S1: Gastric cancer patients' information

Patient Number	Age	Tumor sites	Histology	TNM stage
GC#1	48	Cardia	Intestinal	II
GC#2	62	Non-cardia	Intestinal	I
GC#3	38	Cardia	Diffuse	II
GC#4	59	Cardia	Intestinal	I
GC#5	68	Non-cardia	Diffuse	I
GC#6	64	Cardia	Diffuse	III
GC#7	56	other	Unknown	III
GC#8	36	Non-cardia	Unknown	II
GC#9	34	Cardia	Unknown	II
GC#10	46	Non-cardia	Diffuse	III
GC#11	45	other	Diffuse	III
GC#12	57	Cardia	Unknown	I
GC#13	68	Cardia	Unknown	I
GC#14	72	other	Diffuse	II
GC#15	58	Non-cardia	Unknown	III
GC#16	76	Cardia	Unknown	III
GC#17	79	Cardia	Diffuse	III
GC#18	56	Non-cardia	Unknown	II
GC#19	49	Cardia	Intestinal	II
GC#20	82	Non-cardia	Diffuse	III
GC#21	67	Non-cardia	Unknown	III
GC#22	65	Cardia	Intestinal	I
GC#23	69	Non-cardia	Diffuse	II
GC#24	56	other	Unknown	II
GC#25	46	Cardia	Intestinal	II
GC#26	34	Non-cardia	Unknown	III
GC#27	32	Cardia	Unknown	III
GC#28	65	Non-cardia	Intestinal	III
GC#29	65	Non-cardia	Diffuse	I
GC#30	69	other	Diffuse	II

Tumour, node and metastasis (TNM) stage; other include Site overlapping, Unspecified and Unknown.

Table S2: Comparison of 6PGD mRNA value, protein value and activity level in gastric cancer tissue and normal tissue (Tumor/normal tissue)

Patient Number	mRNA level (fold change)	Protein level (fold change)	Activity level (fold change)
GC#1	3.5	1.4	1.2
GC#2	2.3	1.9	2.2
GC#3	4.0	2.6	2.5
GC#4	1.8	2.1	2.1
GC#5	1.4	1.6	1.8
GC#6	1.2	1.8	2.4
GC#7	3.0	0.9	1.1
GC#8	3.5	3.1	3.1
GC#9	2.2	2.8	3.5
GC#10	3.0	2.6	2.8
GC#11	2.1	2.3	2.3
GC#12	3.3	1.4	1.2
GC#13	1.4	1.5	1.1
GC#14	1.1	2.0	1.1
GC#15	0.9	1.1	0.9
GC#16	1.1	1.1	0.8
GC#17	0.9	1.1	0.8
GC#18	1.5	1.4	2.7
GC#19	2.3	3.4	3.8
GC#20	2.1	2.9	2.8
GC#21	2.3	3.4	3.6
GC#22	1.6	2.7	3.8
GC#23	1.8	2.9	2.9
GC#24	3.0	3.2	3.3
GC#25	1.4	1.8	1.5
GC#26	1.9	2.2	2.2
GC#27	1.2	1.2	0.9
GC#28	2.0	2.3	1.7
GC#29	1.4	2.0	1.4
GC#30	1.4	2.3	2.9
